# A meaningful everyday life experienced by adults with acquired neurological impairments: A scoping review

**DOI:** 10.1371/journal.pone.0286928

**Published:** 2023-10-25

**Authors:** Lena Aadal, Michele Offenbach Hundborg, Hanne Pallesen, Randi Steensgaard

**Affiliations:** 1 Hammel Neurorehabilitation Centre and University Research Clinic, Regional Hospital Central Jutland, Hammel, Denmark; 2 Department of Clinical Medicine, University of Aarhus, Aarhus, Denmark; 3 Specialized Center for Brain Injury, Central Denmark Region, Aarhus, Denmark; 4 Specialized Hospital for Polio and Accident Victims, Aarhus, Denmark; University Hospital Cologne: Uniklinik Koln, GERMANY

## Abstract

**Objective:**

This scoping review explores the characteristics of a meaningful life appraised by adults living with an acquired neurological impairment.

**Introduction:**

Limitations in function, activity or participation following a neurological injury or disease imposes comprehensive changes on the every-day life of the affected person and close relatives. Including patients’ perception of a meaningful life is pivotal to facilitate motivation and individualize rehabilitation efforts to address the patients’ wishes, hopes, needs, and preferences. Surprisingly, only little research has been devoted to illuminating what a meaningful life is from the impaired person’s perspective. Hence, a scoping review of existing knowledge is needed to facilitate person-centered high-quality rehabilitation and research initiatives.

**Inclusion criteria:**

All studies, published in English or Scandinavian languages describing a meaningful life as experienced by adult persons with neurological impairment were included. No search date range filter was selected.

**Methods:**

This review followed the Joanna Briggs Institute (JBI) methodology for scoping reviews according to a published protocol. A three-step search strategy was conducted in the databases PubMed, Cinahl, PsycINFO and Embase. At least two independent researchers conducted inclusions and exclusions, data extraction, and analyses. Covidence software was used to manage the information.

**Findings:**

We identified 307 studies. Of these, 20 were included and quality assessed. Findings are reported in accordance with the PRISMA- SCR checklist and descriptively presented mapped in three main domains and 10 ten sub-domains.

**Conclusion:**

Current literature conveys no clear definition or perception of what a meaningful life is. However, across the 20 included studies, the following main characteristics were stepped forward as particularly significant for adults living with an acquired neurological impairment in regard to achieving a meaningful life: i) to be part of meaningful relationships and meaningful activities; ii) to become confident with one’s perceived identity.

## Introduction

Limitations in function, activity, or participation following a neurological disease or injury impose extensive changes on the everyday life of the affected person and any close relatives [1–3]. Several negative and severe psycho-social consequences have been reported, including reduced quality of life, self-determination, participation and autonomy, weak labor market attachment, and risk of stigmatization [4–10]. These consequences are accompanied by conditions such as physical impairment, reduced physical mobility, and decreasing activity that may further inhibit return to former valued everyday activities [11].

Rehabilitation aims at helping a person to adjust and redefine everyday activities following neurological impairment [12]. When adjusting activities to new realities, it is pivotal to include patients’ wishes, hopes, and preferences in adjusting activities to new realities [13–15] in order to involve, engage, and motivate them to participate in highly intensive rehabilitation programs [16]. This is also central to the outcome of the rehabilitation trajectory [17, 18].

According to the World Health Organization (WHO), rehabilitation is defined as:

*“(…) appropriate measures*, *including through peer support*, *to enable persons with disabilities to attain and maintain their maximum independence*, *full physical*, *mental*, *social and vocational ability*, *and full inclusion and participation in all aspects of life”* [19, 20].

In this definition, the WHO does not explicitly address a meaningful life. The International Classification of Functioning, Disability and Health (ICF) forms a framework for clinical practice. However, it is a biomedical construct of the body with little focus on subjectivity which is a restricting factor in identiying what constitutes meaning and engagement for patients. Guidelines for e.g., post-stroke rehabilitation state that patients should participate in training that is meaningfull, engaging, progressively adaptive, intensive, task specific, and goal oriented. Furthermore, the need for active patient inclusion and participation in all aspects of life is emphasized. Meyer et al. [2020] problematized the lack of a unified definition of rehabilitation, especially in relation to the aim of rehabilitation as the optimization of functioning, especially participation, self-determination, and quality of life, as experienced by the individual [21]. In the Danish healthcare system, the personal perception and experience of meaningfulness lies at the core of rehabilitation; and experiencing a meaningful life is described as the end point of the rehabilitation process [22–24]. Meaningful life thus seems to be an import concept to explore.

Even though the experience of a meaningful life is essentially subjective, it is important to investigate the extent to which citizens with acquired neurological impairment share perspectives on what meaningfulness constitutes. If we can find any such trends, we may support the rehabilitation process.

Several studies claim that person-centered rehabilitation focusing on the patient’s perception of what is a meaningful life improves the outcomes and quality of life for the individual [25–29]. This might be explained by the person’s engagement in his/her own rehabilitation process, leading to individualization according to his/her individual’s wishes, hopes, and needs [26].

Surprisingly, little is known about what people with neurological impairments perceive as meaningful even if obvious improvements in outcome may be achieved by including their preferences. Hence, the patient´s perspective of a meaningful life remains scarcely reported in the literature. Therefore, a scoping review is needed to map and describe what is known about the contents of the goal of rehabilitation–a meaningful life.

## Materials and methods

The aim of the study was to identify how a meaningful life is perceived by adults with acquired neurological impairments. We followed the Joanna Briggs Institute (JBI) methodology [30] and conducted the study according to a published protocol [31].

### Eligibility criteria

We included studies published in English or Scandinavian language describing first-hand experiences of persons with an acquired neurological impairment aged 18 years or older. Quantitative and qualitative designs were considered for inclusion. Qualitative approaches, including, but not limited to, phenomenology, grounded theory and ethnography were also considered.

### Search strategy

The aim of the search strategy was to identify published evidence on the topic. An extensive search was performed in February 2021 and updated in November 2022. As suggested by the JBI, an initial and limited search of PubMed, CINAHL, PsycINFO, and Embase was undertaken to identify articles on the topic. The index and text words contained in the titles were used to develop a full search strategy in collaboration with a university hospital librarian (S1 Appendix). The full search strategy, including all identified keywords and index terms, was adapted for each included database and/or information source. The reference list of all included sources of evidence was screened for additional studies.

### Data extraction process and critical appraisal

Following the search, we uploaded all identified citations uploaded to EndNote and imported them into Covidence software [32] for systematic review management. The review was conducted by an interdisciplinary team of authors, all working within the field of neurological rehabilitation. At least two independent reviewers participated in the iterative process of literature screening, paper selection, data extraction, and analysis. We extracted data using a pre-defined form. Disagreements between the reviewers were resolved by discussion until consensus was reached or through consultation with the entire research team. Firstly, titles and abstracts were assessed to determine if the studies complied with the inclusion criteria. Secondly, full text of selected citations was assessed in detail against the inclusion criteria. An updated search was conducted in December 2022. Seventy-three articles were imported and 20 duplicates removed. Assessment of title and abstract of 53 citations resulted in 51 exclusions. The remaining two sources were full-text screened and one was included but turned out to be a later publication of an earlier included text.

**Fig 1:**
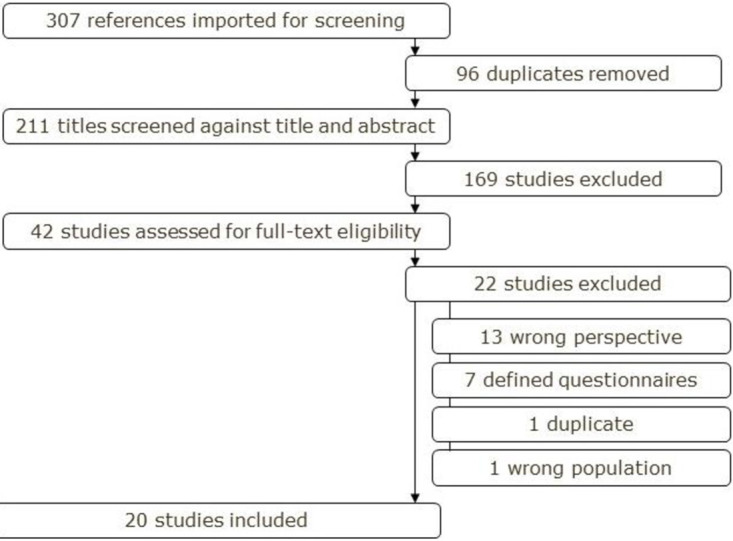
Preferred Reporting Items for Systematic Reviews and Meta-analyses extension for scoping review (PRISMA-ScR) flow diagram.

The results of the search and the study inclusion process are reported using the Preferred Reporting Items for Systematic Reviews and Meta-analyses extension for scoping review (PRISMA-ScR) flow diagram [33] (Fig 1).

Included studies were evaluated using the Critical Appraisal Skills Program (CASP) checklist [34]. Furthermore, a data extraction template was developed and pilot tested by the reviewers. This contributed to a systematic data collection and analysis.

### Data analysis and presentation

Using the Covidence software [32] and the developed tool we extracted the following data from the articles: citations (authors, title, publication year, and journal), country (of origin), sample size, study design, population, study aim, and theoretical foundation. Only published data were extracted. All authors participated in the analysis of the selected articles and the summarizing of the results. To increase trustworthiness of the analysis, we used investigator triangulation to discuss data extraction and findings [35]. An inductive, individual reading of all articles determined the presence of central words and domains. The main coding of domains was presented and discussed amongst all four authors and included only when consensus was reached. The relationships between the domains were also discussed until consensus. A thematic framework for presentation was constructed, and the results are reported narratively according to the identified main themes. We used the PRISMA Extension for Scoping reviews (PRISMA-ScR) Checklist for quality control of scoping reviews [33].

## Results

**Fig 2:**
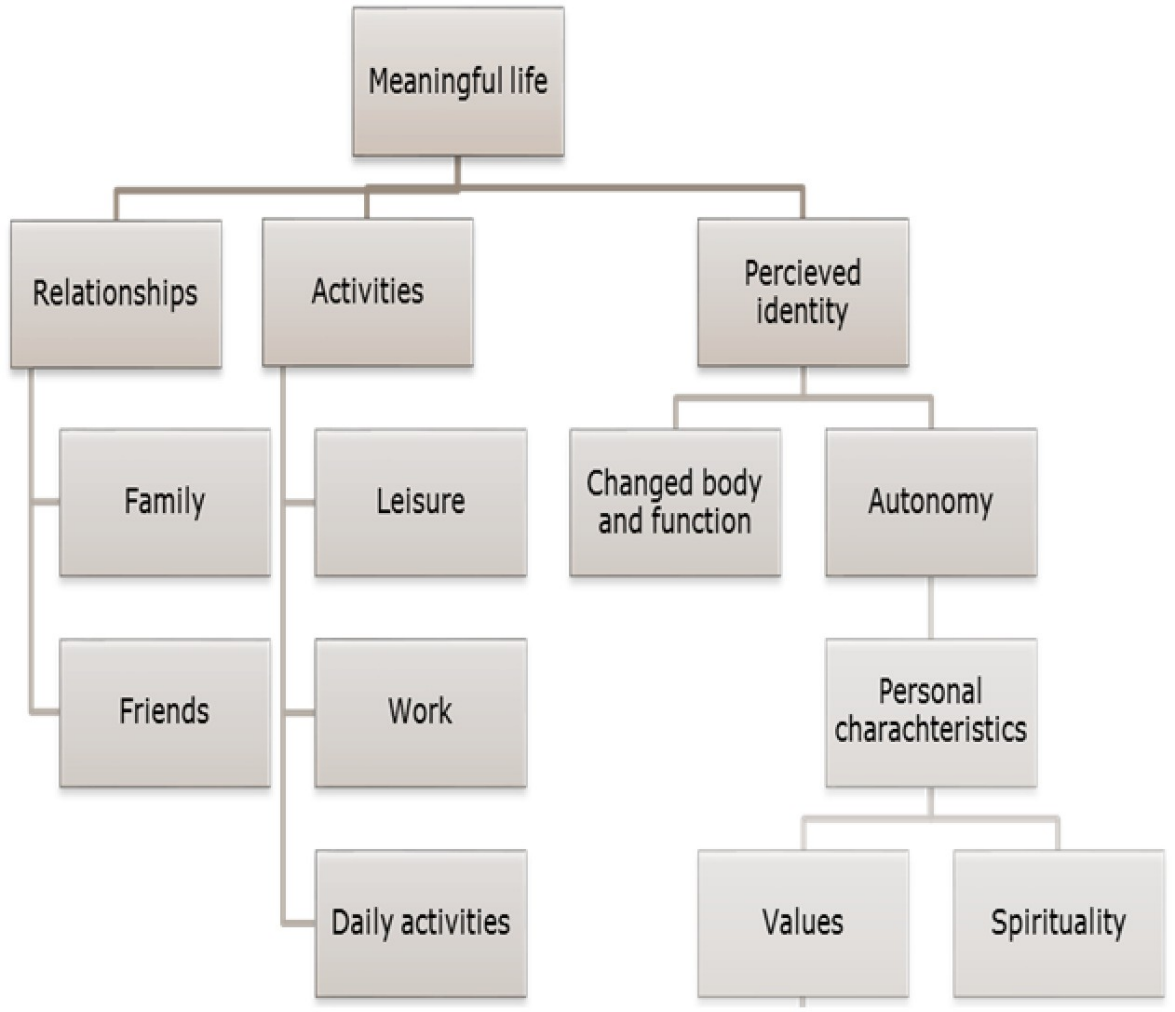
Themes in the analytical process—Meaningful life.

The characteristics of the included studies: As far as country of origin was concerned, one study was from Taiwan, one from Hong Kong, and one from Denmark. Three studies were from Australia, The Netherlands, and the United States; and, finally, four studies were from both the United Kingdom and Canada. As far as study design was concerned, 17 studies applied a qualitative study design, one was a survey, one a mixed methods study combining interviews and questionnaires, and one was a case report. In two studies, the study population were patients with dementia or cerebral palsy, respectively; patients suffered from traumatic brain injury in three studies, multiple sclerosis in three studies, and spinal cord injury in four studies. The most frequently studied population was patients with stroke—eight studies (Table 1 in S2 Appendix). Quality assessment using the Critical Appraisal Skills Program (CASP) checklist [34] indicated poor or moderate quality of the included studies. Four studies had less than seven of ten CASP items fulfilled, while 16 studies fulfilled seven, eight, or nine items. The results of the quality assessments are presented in Table 2 of S3 Appendix. From the mapping process, we found central domains showing the magnitude and importance of "being part of something", and "to become confident with one’s perceived identity" appeared as an important domain during the analysis of how people living with a neurological illness described a meaningful life. Hence, "being part of meaningful relationships" and "being part of meaningful activities" showed to be separate, independent and yet interrelated, consistent domains. Participating in meaningful activities and having life roles and relationships can shape a person’s sense of self, his/her identity, self-image, and self-esteem [36]. The following section will describe the main domains and sub-domains illustrated in Fig 2.

### Mapped domains overview

#### Relationships

Being part of meaningful relationships was a very important source of support for persons with neurological impairments. The relationships include being part of family [2, 37–43] and having friends [2, 36, 39, 41, 43–45].

#### Family

For persons with neurological impairments, family support was an important resource in the recovery process [2, 37, 43]. Social relations with family stimulated the individuals to struggle and survive [37, 40]. As one person with spinal cord injury (SCI) said: *"Considering my kids were so young*, *I could only live on*. *Every time I saw my kids*, *it made me want to fight*. *My family also encouraged me to always hold on*. *Family support is my strength"* [37, p. 317]

Persons with neurological impairments also described how their family relationships changed after the injury [41]. Patients became more aware of their appreciation of their family and to a greater extent prioritized time with them [40]. The family relations seemed to be even more important to the person with traumatic brain injury (TBI), as relationships with others were the most frequently reported source of life meaning [36, 39, 42, 43].

#### Friends

Like family, friends were also considered as an important determinant for neurologically impaired persons living a meaningful life [43, 44]. Patients with TBI expressed how their friends contributed to their feeling of social participation and gave them a positive sense of hope and self-worth [39]. Therefore, friends’ social support had a positive impact on mental and physical health of people with a neurological condition or injury and contributed to heighten their general well-being [2, 39, 44].

However, many persons with neurological impairments experienced that their social network got smaller after the accident. As two TBI patients said: "*Most friends dropped off after the accident*" and "*(*..*) I’ve been a bit of a loner since the accident*" [36, p. 1277]. Absence of support from friends increased their susceptibility to psychological distress because it could permeate their sense of self and disrupt their identity and self-image [36, 39]. Interestingly, patients’ connection to friends cannot be replaced by family members. Thus, patients told that when only family members covered friendship functions, this restricted their ability to share thought, feelings, and problems [39].

Summing up, being part of meaningful relationships is closely related to living a meaningful life. Social relationships with both family and friends are important because the social relations contribute differently to the patient’s life when the patient has to recover from a neurological impairment.

#### Activities

Despite being of a lower rank, employment, education, and valued hobbies were also important domains in sustaining self and social status and a valuable life [38–41]. Hence, meaningful activities including work life [38–40, 46], leisure activities [42, 47, 48], and activities of daily living [49, 50] were experienced as important in relation to achieve a meaningful life [38–41].

#### Employment

For persons with TBI, work was important to their sense of self, just as being able to work affected their status within social groups [38]. Work was a place that helped them sustain and develop friendships [38]. For persons living with multiple sclerosis (MS), work remained a meaningful activity as a means of staying active and retaining one’s identity, social role, and sense of purpose and direction in life [51]. Nevertheless, it was no longer as important as before the disease. Having realized that it was no longer realistic to strive for a higher position and to make long-term career plans, they adjusted their expectations and re-familiarized themselves with their own body to be able to continue their work [46]. This required social and emotional processing and conscious decisions about life activities, including the work that had to be done to restore balance [46] people impaired by neurological illness, taking part in leisure activities was closely related to a meaningful life. Leisure was described as a key activity in life, connecting growth and meaning making in pursuit of a purposeful life [42, 47]. The overarching theme associated with leisure was engagement with life, providing the person with a feeling of living a fulfilling, engaged, and enriched life through their active engagement with self, others, and nature/world [47]. Furthermore, engagement through leisure may further the experience of living a composed and joyful life and can provide self-identity, self-discovery, and connectedness in life (e.g., socially, spiritually) [47]. The sense of connectedness can be social, spiritual, and cultural; and it and has the potential to strengthen interpersonal, social relationships. Leisure seemed to provide less restrictive and more flexible and liberating opportunities (than, e.g., work) to adjust or change the pace of life, so as to live life in a more harmonious, balanced way. Leisure activities may even help persons cope with stress, further healing, and facilitate growth and transformation; thereby providing resilience and empowerment in a context promoting personal transformation [47].

When losing the possibility to participate in prior valued leisure activities on equal terms, like before disease struck, people felt that they became more of a burden. As explained by Eve in the study by Purton, Sim, and Hunter (2021) [36], ballroom dancing was no longer possible because “[…] *you can’t really jive with just one hand–it’s two hands*” [36]. Similarly, persons with congenital physical disabilities described how engaging in leisure activities had mental and physical health benefits while providing enjoyment, helped them prove themselves, led to friendship building, and gave them a sense of belonging [48].

#### Daily activities

Daily activities occur and take place in an arena where role management and a meaningful life are co-constructed and negotiated with others. The value of participation and being able to perform sense-making everyday activities was described as follows: "[…]*the activities participants spoke about were–e*.*g*. *gardening*, *looking after animals*, *and cooking"* [49]. For stroke survivors and their spouses, *‘everyday activities became an empowering or incompatible mirror of the stroke survivor’s capacities in daily situations’* [50]. Being able to manage daily activities again gave a sense of independence and confidence [49].

Hence, patients with stroke felt that it was important to visit their home in the weekends during hospitalization [49]. Accordingly, activities such as gardening, cooking, or looking out for animals gave stroke survivors a sense of purpose along with confidence and independence [49].

"Being part of meaningful relationships" and "Being part of meaningful activities"

Being part of social networks facilitated through work and leisure activities resulted in shared living arrangements providing close relationships that contributed to wellbeing and maintenance of a positive sense of self-worth [39]. Similarly, participation in activities provided a sense of engagement and inclusion [36, 49]. Closeness and contributing relationships with others in general were essential [39]. However, the close family life with spouses, children, and loved ones was described as absolutely crucial to constitute a self and to sustain a meaningful life after the injury [38, 40].

#### Perceived identity

Perceived identity was another important domain that appeared during the analysis of how people living with a neurological illness described a meaningful life. Furthermore, this theme was categorized into at least two sub-domains: Changed body and function, and autonomy.

#### Changed body and function

Physical dysfunctions seriously impacted the self-image and self-esteem of persons with SCI [37]. Adaptation to a new meaningful life was described to be composed of two stages: (a) mourning the past and trying to accept physical disabilities; and (b) self-acceptance and growth [37]. The appearance of and perceived attitudes towards disabilities caused shame and disgust and affected their adaptation to their new situation, making it difficult to re-integrate into society [37]. In another SCI study [44], changes were described as circumstantial, as seen in quotations like the following from a Moroccan man (42, single, Muslim, high school, paraplegia, incomplete): *“In my situation*, *the way I know myself*…*(own name) is still (own name)*. *Nothing different about that*. *Yes*, *I have a*…*I am (own name) with a little extra”* [44]. The authors concluded that much had changed in his life but not his identity despite the SCI`s influence on overall meaning of life [44].

For stroke survivors, the central process of adaptation to a new life was described as reconstruction of an occupational identity, facilitated through connections within and across three domains–self, others, and reality [52]. Connecting with self-involved emotional management, motivation, confidence, occupational engagement, and seizing control. Connecting with others included being understood, belonging, receiving help, and engaging in interactions. Connecting with reality meant confronting the impact on daily life and one’s unfolding life story according to three realities: past reality, the reality of the stroke, and future reality [52]. People with stroke, aphasia, or upper limb dysfunction felt that their dysfunction prevented them from participating in meaningful activities; and these impairments therefore had a very evident impact on their sense of self [36, 49].

In a dementia study [53], the metaphorical description of moving from ‘up there’ to ‘down here’ represented the perceived struggle to maintain a sense of worth despite a marginalized social position and reduced meaning in everyday life. In this paper, finding meaning was described to involve looking backwards to sustain continuity with the past and looking forwards to maintain momentum and keep going [53].

For all these participants across the neurological field, the changed body and function affected their autonomy and perceived identity.

#### Autonomy

People with stroke [2, 36, 43, 45, 49, 52], SCI [37, 44], TBI [38, 40], MS [42], and dementia [53] had experienced change in autonomy to influence the global meaning in the process of adapting to a new life. It seems to be a universal issue when dealing with illnesses across the neurological field. In the above-mentioned studies, we found no clear theoretical rationale or description of how change, perceived identity, or autonomy affected meaningful life. It seems that people become more aware of aspects of their global meaning, when they are confronted with vulnerability, loss of ability, or changed appearance. ’More in control of their recovery and aware that along the recovery path they had to ‘reinvent themselves, they were more successful at reengaging in social participation’ [2]. Furthermore, quality of life is a dimension of meaning associated with maintaining a sense of social worth. Finding meaning involves looking backwards to sustain continuity with the past and looking forwards to maintain momentum and keep going [53].

However, another sub-theme steps forward as an essential aspect in the analysis: Personal characteristics—meaning a kind of "personal approach" of how to handle (difficult) life situations.

#### Personal characteristics

Through the process of mapping emerged characteristics such as will power, mood, motivation, positive thoughts, and taking control in own life. Positive acting facilitates successful living [49] Klik eller tryk her for at skrive tekst., *then*, *I am being a burden*" [2], and the authors concluded ’participants who had set goals for themselves reported greater confidence in getting back to doing the things they enjoyed’ [2]. Therefore, being able to take initiative and having control over one’s life appear meaningful.

Participants in a MS study generated evocative descriptions of resilience: ’*Psychological adaptation*, *social connection*, *life meaning*, *planning*, *and physical wellness emerged as facilitators* [42]. Resilience depletion, negative thoughts and feelings, social limitations, social stigma, and physical fatigue emerged as barriers to resilience [42]. Most participants in a TBI study tried to create a positive and meaningful existence by using what Folkman [54] described as "positive reappraisal". The authors summarized that regardless of the severity of their injury, all participants needed to find positive meaning in their stressful situation [40].

Personal characteristics, the way you act and interact with others, and especially the fact that you behave or embrace the world positively seemed to have an impact on experiencing being an actor in a meaningful life. This is in line with our mapping of sub-domains containing personal values and beliefs which will described narratively in the following section.

#### Changing values

A change in personal values following a neurologic disease was described in nine studies (stroke in 7, [49]; TBI in [38–40]; SCI in [44]; dementia in [53], MS in [42], and SCI/ABI in [41]). Persons suffering from stroke perceived that their subjective understanding of the consequences of their illness for their everyday life influenced their mood and life satisfaction [55]. In relation to their everyday life activities, the consequences were experienced as fundamental during the process of obtaining a changed self and an understanding that life could go on [49]. Some patients suffering from TBI "*were provoked by the illness to reassess their values as the injury became a symbol of a new beginning*" [40]. After the injury, they appreciated that things were not important before [39, 40]. After their injury, they no longer took their life for granted, appreciated the human body and mind, and had become more aware of their own morbidity [40]. Most of the core values centered around appreciation of life, their abilities and independence, and awareness of their own vulnerability [41]. However, new aspects appeared regarding the understanding of human diversity and perceived close human relations and they had come to better understand and appreciate the importance of relations [41].

#### Spirituality

Two studies described aspects of spirituality in everyday life with a neurologic disease–one among persons with SCI [44], the other in patients with SCI or ABI [41]. Persons with SCI referred to God’s will in order to create meaning in their changed life. A patient explained: "*Whom god loves*, *he chastises*" to explain horrible facts in life [44].

In patients with ABI or SCI, spirituality seemed to interconnect religion and meanings in life [41]. A patient experienced a different outlook on God and perceived his illness as His will: *"God left me on this earth to serve a purpose—This is my second chance and I better not screw it up"* [41]. It gave them the opportunity to improve their situation by being a better fellow human being. This resulted in changed values and a new sense of purpose in life [41].

## Discussion

This paper presents the first systematic and comprehensive review of current literature on how a meaningful life is perceived by adults with acquired neurological impairments. We excluded grey literature, such as dissertations, conference proceedings, and reports and documents on organizational websites. The included studies were situated in various contexts, and the majority were conducted after the patients’ discharge. The studies were conducted all over the world except on the African continent. Hence, we suggest that this scoping review gives the best possible global perspective on a meaningful life following acquired neurological impairment.

A range of factors collectively contributed to a meaningful life for persons with neurological impairments. These factors included relationships to friends and family; activities like leisure, work, daily activities; and perceived identity. We found that the domains were separate, independent, and yet interrelated; each with a number of subdomains which will be discussed in the following. The domains of meaningful life explored in this review can be understood as belonging to an iterative process. This process involves aspects of “being, doing, belonging, and becoming” [56–58];and they are fundamental aspects of being a human being. Thus, in a Scandinavian study [59], Pedersen et al. provided an understanding of essential human desires and possibilities significant for quality of life. In this study [59], they found that being was linked to doing (action or engagement), belonging (relationships and connectedness), and becoming (a perceptual process of change and development) [59]. Several factors threatened reconstruction of meaningful life.

In the present scoping review, we found that the participants’ perceived capacity and identity together with various personal ways of handling and overcoming the often persistent consequences of their ailment seemed to influence their ability to live a meaningful life. However, doing activities and engaging in work and leisure were described to have a positive effect on life’s meaningfulness; furthermore, having relationships, friends, and family gave persons with acquired neurological impairments a sense of belonging. Although it was not directly expressed by the participants in the 20 included studies, it seems obvious that succeeding in being, doing, and belonging (work, leisure, activities, friends and family) can facilitate a transition back to “normality”, influencing their experience of life’s meaningfulness.

We found that relationships are strongly related to living a meaningful life for persons with neurological impairments. It was clear that social relations with family and friends were an important resource for the person in the recovery process, and that these close social relations stimulated the person’s will to fight and survive. This is in line with a basic human need to engage in close social relationships and to ’belong’ with others [60].

Doing activities and engaging in work and leisure were described to have a positive effect on life’s meaningfulness. This is in line with positive psychology where significance in life and a purpose or overarching aim in life seems fundamental [61, 62]. The experience of meaning in life involves perceptions that every day experience is causally and temporally coherent and that changes results from activities related to self-development, i.e. a sense of self efficacy and contribution to others, i.e. developing and maintaining close relationships [63]. In other words, the meaning of coherence is associated with "the feeling that one’s experience or life itself makes sense" [64 p. 9]. Meaningfulness is actualized through positive functioning, the enjoyment of work, positive affect, and hope [65].

Perceived identity was another important domain that appeared during the analyses. This might be understood as a fundamental human need to "*be who we are by manifesting our individuality and to be seen*, *appreciated*, *understood*, *and mirrored in relation to our self-presentation and self-assertion"* [66 p. 5]. We found that the constitution of identity was related to the autonomy in fulfilling roles and tasks in daily life. This facilitated motivation and a willingness to fight to obtain the best possible functioning. This is in line with the need for competence and concerns the individual’s *"need to develop competences such as abilities*, *talents*, *and skills to master our natural*, *personal*, *and social worlds*. *This is thus about ’mastering my tasks"* [66 p.5]. In other words, persons with acquired neurological impairment strove to achieve a high degree of autonomy in managing as many tasks in their daily life as possible, and thus experience coherence and a sense of mastery. In this struggle, some persons with SCI and ABI found support in spirituality, and they used their increased religiosity to handle difficult situations. This might be understood as a way to understand one’s existential being in the world [66]. As reconstruction of meaning is essential for living a meaningful life, it is surprising that spirituality was addressed in only two studies, illustrating that further research is needed.

Based on the above, the map of domains related to a meaningful life shows items related to life aspects rather than aspects of disease or impairment. Thus, as discussed, the domains seem aligned with man’s basic psychological needs [66], such as the need for meaning [67], relatedness, autonomy, and competence [60]. This supports the use of a biopsychosocial and holistic approach suggested for rehabilitation by both the WHO (ICF model) [68] and the established definitions of rehabilitation; all perceiving participation as a core concept [24, 69]. This implies rehabilitation trajectories influenced by personal values and habits. Furthermore, there is a strong need for exploring the contents of meaningful life. This issue should be explored in the context of the recent definition of rehabilitation, viz. "*to enable a meaningful life with the best possible activity and participation*, *coping and quality of life*" [24].

Unfortunately, rehabilitation efforts are challenged by limitations of the existing knowledge base, especially concerning broad aspects of quality of life, autonomy, and the meaningfulness of the lived life [70–72]. Hence, our findings implicate an urgent need for further research in order to address the patient’s rehabilitation needs in regard to a meaningful life [73].

### Limitations

Despite a comprehensive search strategy using the most relevant databases and an experienced librarian, the primary limitation of this study was the ambiguous definition of what constitutes a meaningful life. Hence, a risk exists that relevant published literature did not emerge from the databases despite our search strategy. However, the identification of 96 duplicates indicates adequate density of the search. Additionally, the variability in the nature of research methods used in the included papers presented a challenge for the narrative presentation of the results of this review. Although it is not a requirement in scoping reviews, this review assessed the quality of the included studies to improve the transparency in our findings. Extractions from the included reviews were critically evaluated for interpretation by all authors using the original studies. The credibility of our review was improved by using an established method [30, 33, 74], and trustworthiness was increased by investigator triangulation, resulting in valuable discussions and transferable interpretations of our findings [35].

## Conclusion

The literature offers no clear definition or perception of what a meaningful life is. However, across the 20 included studies, the following main characteristics emerged as significant for adults living with an acquired neurological impairment in achieving a meaningful life: i) to be part of meaningful relationships and meaningful activities; and ii) to gain confidentiality with perceived identity. The domains of meaningful life have similarities with general basic human psychological needs and can be understood as being part of an ongoing iterative process involving “being, doing, belonging and becoming”.

## Supporting information

S1 Appendix Search strategy. (DOCX)

S2 Appendix Characteristics of included studies, Table 1. (DOCX)

S3 Appendix Results of the quality assessments of included studies, Table 2. (DOCX)

S1 File (PDF)
