# Supplementary material for: A meaningful everyday life experienced by adults with acquired neurological impairments: A scoping review
Source: PLoS One. 2023 Oct 25;18(10):e0286928. doi: 10.1371/journal.pone.0286928 (PMC10599513; doi:10.1371/journal.pone.0286928)
Supplement: S1 Appendix — (DOCX) [file pone.0286928.s001.docx]

**Pubmed**

("Nervous System Diseases"[MeSH Terms] OR ("brain injur*"[Text Word] OR "spinal cord trauma*"[Text Word] OR "spinal cord injur*"[Text Word] OR "spinal cord transection*"[Text Word] OR "spinal cord lacer*"[Text Word] OR "traumatic myelopath*"[Text Word] OR "spinal cord contusion*"[Text Word] OR "stroke*"[Text Word] OR "apoplex*"[Text Word] OR "cerebrovascular accident*"[Text Word] OR "brain vascular accident*"[Text Word] OR "multiple sclerosis"[Text Word] OR "disseminated sclerosis"[Text Word] OR "brain laceration*"[Text Word] OR "parkinson*"[Text Word] OR "paralysis agitans"[Text Word] OR "amyotrophic lateral sclerosis"[Text Word] OR "lou gehrig disease*"[Text Word])) AND ("Rehabilitation"[MeSH Terms] OR "rehabilitat*"[Text Word]) AND ("Sense of Coherence"[MeSH Terms] OR ("meaningful life"[Text Word] OR "meaning in life"[Text Word] OR "meaning of life"[Text Word] OR "life meaning*"[Text Word] OR "meaningful everyday life"[Text Word] OR "meaningful living"[Text Word])) AND ("danish"[Language] OR "english"[Language] OR "norwegian"[Language] OR "swedish"[Language])

**Cinahl**

((MH "Nervous System Diseases+") OR (MH "Spinal Cord Injuries+") OR "brain injur*" OR "spinal cord trauma*" OR "spinal cord injur*" OR "spinal cord transection*" OR "spinal cord lacer*" OR "traumatic myelopath*" OR "spinal cord contusion*" OR "stroke*" OR "apoplex*" OR "cerebrovascular accident*" OR "brain vascular accident*" OR "multiple sclerosis" OR "disseminated sclerosis" OR "brain laceration*" OR "parkinson*" OR "paralysis agitans" OR "amyotrophic lateral sclerosis" OR "lou Gehrig disease*") AND ((MH "Rehabilitation+") OR Rehabilitat*) AND ((MH ”Life Purpose”) OR "meaningful life" OR "meaning in life" OR "meaning of life" OR "life meaning*" OR "meaningful everyday life" OR "meaningful living") AND LA (danish or english or norwegian or swedish)

**Embase**

(exp neurologic disease/ or (brain injur* or spinal cord trauma* or spinal cord injur* or spinal cord transection* or spinal cord lacer* or traumatic myelopath* or spinal cord contusion* or stroke* or apoplex* or cerebrovascular accident* or brain vascular accident* or multiple sclerosis or disseminated sclerosis or brain laceration* or parkinson* or paralysis agitans or amyotrophic lateral sclerosis or lou gerig disease).mp.) and (exp rehabilitation/ or "rehabilitat*".mp.) and ("sense of coherence"/ or (meaningful life or meaning in life or meaning of life or life meaning or meaningful everyday life or meaningful living).mp.) and (danish or english or norwegian or swedish).lg.

**PsycINFO**

(exp nervous system disorders/ or (brain injur* or spinal cord trauma* or spinal cord injur* or spinal cord transection* or spinal cord lacer* or traumatic myelopath* or spinal cord contusion* or stroke* or apoplex* or cerebrovascular accident* or brain vascular accident* or multiple sclerosis or disseminated sclerosis or brain laceration* or parkinson* or paralysis agitans or amyotrophic lateral sclerosis or lou gerig disease).mp.) and (exp rehabilitation/ or "rehabilitat*".mp.) and ("sense of coherence"/ or (meaningful life or meaning in life or meaning of life or life meaning or meaningful everyday life or meaningful living).mp.) and (danish or english or norwegian or swedish).lg.
