# Supplementary material for: A meaningful everyday life experienced by adults with acquired neurological impairments: A scoping review
Source: PLoS One. 2023 Oct 25;18(10):e0286928. doi: 10.1371/journal.pone.0286928 (PMC10599513; doi:10.1371/journal.pone.0286928)
Supplement: S2 Appendix — (DOCX) [file pone.0286928.s002.docx]

| **Ref.** | **Authors, Title,** | **Country, Year** | **Population** | **Sample Size** | **Age**  **Mean (SD)** | **Study Design** | **Aim** |
| --- | --- | --- | --- | --- | --- | --- | --- |
| (37) | *Ching-hui Chuang; Yu-O. Yang; Liang Tseng Kuo*  Finding a Way to Cope: A Qualitative Study | Taiwan,  2015 | Persons with SCI | 10 participants (8 M, 2 F) | 24-43 | Qualitative research. | To explore the lived experiences of the persons with SCI. |
| (55) | *Chow, E.O. W.*  The role of meaning in life: mediating the effects of perceived knowledge of stroke on depression and life satisfaction | Hong Kong,  2017 | Stroke | 192 elderly (118 M, 73 F) | 72.67 (7.53) | Survey. Baseline data from an RCT. Correlation and mediation analyses of mediating effects of meaning of life/knowledge/life satisfaction were conducted | To investigate the significance of survivors’ meaning ofin life in relation to their perceived knowledge and psychological wellbeing. |
| (38) | *Conneeley, A. L.*  Transitions and brain injury: : A Qualitative Study Exploring the Journey of People with Traumatic Brain Injury | UK, 2012 | TBI | 18 cases of TBI person (5 F), significant other, team-members | Mean 35 | A longitudinal, phenomenological approach. Semi-structured interviews. | To explore transitions from hospital to home over a period of 1 year. |
| (39) | *Douglas J*  Loss of friendship following traumatic brain injury: A model grounded in the experience of adults with severe injury | Australia, 2019 | TBI | 23 (20 M, 3 F). On average, 10 years had elapsed since the injury | 25–45 | Convergent mixed methods (quantitative measures and in-depth interviews). Qualitative analysis of interview transcripts moved through a process of data-driven open and focused coding (gGrounded theory). | To understand the post-injury experience of friendship from the perspective of adults with severe TBI. |
| (40) | *Graff, H.J.; Christensen, U; Poulsen, I; Egerod, I*  Patient perspectives on navigating the field of traumatic brain injury rehabilitation | Denmark,  2018 | TBI | 20 participants (12 M , 8 F) with a mild, moderate, or severe TBI | 25–63 (39) | Qualitative explorative design using semi-structured in-depth interviews with TBI survivors at one to four years after post hospital discharge. | To provide an understanding of the lived experience of rehabilitation in adults with traumatic brain injury (TBI) from hospital discharge up to four years post-injury |
| (49) | *Grohn, B; Worrall, L.E.; Simmons-Mackie, Nina; Brown, Kyla*  The first 3-months post-stroke: What facilitates successfully living with aphasia | Australia,  2012 | Stroke | 15 participants with aphasia after stroke, (8 M, 7 F). Severity of aphasia tended to be moderate to mild | 47-90 | Qualitative, phenomenological, sSemi-structured interviews and assessments were completed at 3 months after post-onset. | To explore the perspective of people with aphasia on factors that facilitate successful living. |
| (47) | *Iwasaki, Y.*  Leisure and meaning-making: Implications for rehabilitation to engage persons with disabilities | Canada,  2017 | Disabled people | Summarises research | adults | Qualitative. Guided by the research-based knowledge about the role of leisure in meaning-making. | To identify and map the current research-based knowledge about the role of leisure in meaning-making, and describe its implications for rehabilitation. |
| (44) | *Littooij, E.; Widdershoven, G.A.M.; Stolwijk-Swüste, J.M.; Doodeman, S.; Leget, C. J.W.; Dekker, J.*  Global meaning in people with spinal cord injury: Content and change | Netherlands,  2016 | Persons with SCI | 16 (9 M, 7 F) participants who received outpatient rehabilitation | 26- 79 | Qualitative; grounded theory. Interviews were held between 6–24 months after admission to the rehabilitation centre. Loosely structured from topic list. | The purpose of this study was twofold: (i) to explore the content of global meaning of people with SCI, and (ii) to explore whether or not global meaning changes after SCI. |
| (2) | *Masterson-Algar, P.; Williams, S.; Burton, C.R.; Arthur, C.A.; Hoare, Z.; Morrison, V; Radford, K.; Seddon, D.; Elghenzai, S.*  Getting back to life after stroke: co-designing a peer-led coaching intervention | UK,  2020 | Stroke | 18 stroke survivors residing in community settings and 10 family carers |  | Co-design. Phase 1 assessed function, mood, and involvement in leisure and social activities 6 months following stroke. Phase 2 involved semi-structured, in-depth interviews. Phase 3 tested the co-designed peer-led coaching intervention. | Tto co-design and test a novel peer-led coaching intervention that enabled stroke survivors to rebuild meaningful social and leisure activities after stroke. |
| (41) | #78  *McColl M.A.; Bickenbach, J; Johnston, J.; Nishihama, S.; Schumaker, M.; Smith, K.; Smith, M.; Yealland, B.*  Changes in Spiritual Beliefs After Traumatic Disability | Canada,  2000 | Traumatic Disability | 16 individuals (12M,4F), all of whom had: (1) a traumatic-onset, moderate to severe disability, specifically a spinal cord injury(SCI), brain injury, or both; | 20- 80 (37) | Qualitative methodology in a cross-sectional study using semi-structured interviews. | To uncover perceptions about changes in spirituality since the onset of the disability. |
| (51) | Meade, M.; Rumrill, P.; Krause, J.S.; Reed, K.S.; Aust, R.  Perceptions of Quality of Employment outcomes after Multiple Sclerosis. | United States,  2016 | MS | 74 participants at time of diagnosis and employed after diasgnosis | 18-65 | Qualitative approach using eight focus groups at three sites. | To identify quality outcomes of employment, as defined by those with MS. |
| (46) | *Meide, H. van der; Gorp, D. van; van der Hiele, K.; Visser, L.*  Always looking for a new balance": toward an understanding of what it takes to continue working while being diagnosed | Netherlands,  2018 | MS | 19Nineteen (8 M, 11 F) employed adults diagnosed with relapsing-remitting MSmultiple sclerosis. | 29-55 | Narrative interviews | To gain insight into the meaning of work in the everyday lives of people with relapsing-remitting MSmultiple sclerosis. |
| (45) | *Pilkington F.B.*  A qualitative study of life after stroke | Canada, 1999 | Stroke | 13 participants, (9 M, F) | 40-91 | Qualitative research. "Loosely structured interviews aimed a eliciting descriptions of quality of life were scheduled during the acute care stay and a t one and three months after stroke onset". | To enhance understanding about quality of life after a stroke from the patients' perspective. |
| (36) | Purton, J.; Sim, J.; Hunter, S. M.  The experience of upper-limb dysfunction after stroke: a phenomenological study | UK,  2021 | Stroke | 13 adult stroke survivors with upper-limb impairment in a stroke rehabilitation unit | 18 - | Qualitative research. A "a series of up to four semi-structured interviews over an 18- month period after stroke. | To explore stroke survivors’ experiences of upper-limb dysfunction over time. |
| (53) | *Robertson, J.M.*  Finding meaning in everyday life with dementia: a case stud | UK,  2014 | Dementia | One older woman’s perspective on the quality of her life with dementia. |  | Case report. | To understand how she evaluates her changed situation in the present compared to the past. |
| (50) | *Satink, T.; Josephsson, S.; Zajec, J.; Cup, E.H. C.; de Swart, B.J. M.; Nijhuis-van der Sanden, M.W. G.*  Negotiating role management through everyday activities: narratives in action of two stroke survivors and their spouses activities. | Netherlands,  2016 | Stroke | Two stroke survivors and their spouses. | Stroke survivor 67 and 68 | Qualitative research. Pparticipant observations inat their own environment. | To explore how stroke survivors act as role managers with their spouses in the context of everyday activities. |
| (42) | *Silverman, A.M.; Verrall, A.M.; Alschuler, K.N.; Smith, A.E.; Ehde, D.M.*  Bouncing back again, and again: a qualitative study of resilience in people with multiple sclerosis. | United States,  2017 | MS | Four focus groups | 36-62 | Qualitative rResearch.  Focus groups interviews | To describe the meaning of resilience, factors facilitating resilience and barriers to resilience, from the perspective of persons with MSmultiple sclerosis, their care partners and community stakeholders. |
| (48) | *Specht J.; King G.; Brown E.; Foris C.*  The importance of leisure in the lives of persons with congenital physical disabilities. | Canada,  2002 | Cerebral palsy or spina bifida | Nine adults | 30-50 | Qualitative research;  a secondary analysis of the interview protocols of nine adults. | To determine the meaning of involvement in leisure occupations in the patient’´s lives. |
| (43) | *Torregosa, M. B.; Sada, R.; Perez, I*.  Dealing with stroke: Perspectives from stroke survivors and stroke caregivers. | USA/Mexico,  2018 | Stroke | 11 stroke survivors and eight stroke caregivers. | Living with stroke ranged from 1 to 24 years; (average 7 years). | Qualitative exploratory research. Two separate focus groups were conducted, one each for each stroke survivors and caregives. | To examine the lived experiences of post-stroke recovery and readjustment among stroke survivors and stroke caregivers. |
| (52) | *Walder, K.; Molineux, M.*  Re-establishing an occupational identity after stroke - a theoretical model based on survivor experience. | Australia,  2017 | Stroke | Six adult  (2 M, 4 F) | 34-76 | Qualitative research.  Iin-depth interviews using constructivist grounded theory methodology. | To understand occupational disruption and identity reconstruction after stroke. |

Appendix table 1. Characteristics of included studies.
