# Supplementary material for: A meaningful everyday life experienced by adults with acquired neurological impairments: A scoping review
Source: PLoS One. 2023 Oct 25;18(10):e0286928. doi: 10.1371/journal.pone.0286928 (PMC10599513; doi:10.1371/journal.pone.0286928)
Supplement: S3 Appendix — (DOCX) [file pone.0286928.s003.docx]

| **Study /Quality** | 1 | 2 | 3 | 4 | 5 | 6 | 7 | 8 | 9 | 10 |
| --- | --- | --- | --- | --- | --- | --- | --- | --- | --- | --- |
| (37) *Chuang et al.* | yes | yes | yes | yes | yes | no | yes | yes | yes | no |
| (55) *Chow* | not app | yes | yes | yes | yes | not app | not app | yes | yes | yes |
| (38) *Conneeley* | yes | yes | yes | yes | yes | yes | not app | yes | yes | yes |
| (39) *Douglas* | unclear | yes | yes | unclear | unclear | no | no | unclear | unclear | unclear |
| (40) *Graff et al.* | yes | yes | yes | yes | yes | yes | unclear | yes | yes | yes |
| (49) *Grohn et al.* | yes | yes | yes | yes | yes | unclear | no | yes | unclear | yes |
| (47) *Iwasaki* | not app | unclear | unclear | unclear | unclear | no | no | not app | not app | yes |
| (44) *Littooij* | unclear | yes | yes | yes | yes | no | yes | yes | yes | yes |
| (2) *Masterson-Algar et al.* | unclear | yes | yes | yes | yes | no | yes | yes | yes | yes |
| (41) *McColl et al.* | unclear | yes | yes | yes | yes | unclear | no | yes | yes | yes |
| (51) Meade et al. | yes | yes | yes | yes | yes | unclear | unclear | yes | unclear | yes |
| (46) *Meide et al.* | yes | yes | yes | yes | yes | no | no | yes | yes | yes |
| (45) *Pilkington et al.* | yes | yes | yes | yes | yes | unclear | unclear | yes | yes | yes |
| (36) *Purton et al.* | yes | yes | yes | yes | yes | unclear | no | yes | yes | yes |
| (53) *Robertson* | yes | yes | yes | yes | yes | no | no | yes | yes | unclear |
| (50) *Satink et al.* | yes | yes | yes | yes | yes | no | no | unclear | unclear | yes |
| (42) *Silverman et al.* | unclear | unclear | yes | unclear | no | no | yes | yes | no | unclear |
| (48) *Specht et al.* | yes | yes | yes | yes | yes | unclear | Unclear | yes | unclear | yes |
| (43) *Torregosa et al.* | yes | yes | yes | yes | yes | yes | no | yes | yes | yes |
| (52) *Walder et al.* | yes | yes | yes | yes | yes | unclear | unclear | yes | yes | not app |

Appendix table two: Quality assessment of the included studies using the CASP instrument.
