# Supplementary material for: A meaningful everyday life experienced by adults with acquired neurological impairments: A scoping review
Source: PLoS One. 2023 Oct 25;18(10):e0286928. doi: 10.1371/journal.pone.0286928 (PMC10599513; doi:10.1371/journal.pone.0286928)
Supplement: S1 File — (PDF) [file pone.0286928.s004.pdf]

VidKom

This document certifies that the manuscript entitled

A meaningful everyday life experienced by adults with acquired neurological impairments: a scoping review

By

Lena Aadal et al.

was edited for proper English language, grammar, punctuation, spelling, and overall style by one or more of the highly qualified native English-speaking editors at VidKom.

This certificate was issued on **8 May 2023** and may be verified by contacting VidKom at mpi@vidkom.dk.

VidKom
Editing Certified

Neither the contents of the research nor the intentions of the authors were changed in any way during the editing process. Documents receiving this certification should be English-ready for publication; however, the author has the ability to accept or reject our suggestions and make any changes they wish after having received the proofread and copyedited version.

If you have any questions or concerns about the edited document, please contact VidKom at mpi@vidkom.dk
